# Supplementary material for: Postpartum long-acting reversible contraceptives use in sub-Saharan Africa. Evidence from recent demographic and health surveys data
Source: PLoS One. 2023 Oct 9;18(10):e0291571. doi: 10.1371/journal.pone.0291571 (PMC10561849; doi:10.1371/journal.pone.0291571)
Supplement: Attachment — Submitted filename: Response to Reviewers.docx [file pone.0291571.s002.docx]

Dear reviewer, we would like to extend our deepest appreciation for devoting your time to reviewing our manuscript entitled " Postpartum long-acting reversible contraceptives use in Sub-Saharan Africa. Evidence from recent Demographic and Health Surveys Data". there has been a revision of this manuscript. The whole structure of the manuscript has been revised. We hope now the manuscript is clear and more acceptable than its previous version. We have tried to present the paper in the proper manner according to your comment on what to supposed to do so. For this, here we have given our responses to each of the concerns you raised. Again, we would like to remind our strongest gratitude for your effort in the improvement of this manuscript, and all the points were addressed in the point-by-point response.

Reviewer 1

Reviewer comments: Line 45: Secondary data analysis was performed using the recent Demographic and Health Survey (DHS)

Authors response: thank you for your observations and comments, this has been addressed, kindly see line 45.

Reviewer comments: Line 47: A multilevel mixed-effect logistic regression model was used to identify factors associated with long-acting reversible contraceptive use.

Authors response: Dear reviewer thank you for your comments, this has been addressed, kindly see line 46-47.

Reviewer comments: Line 92: Even though the postpartum period is the best opportunity for LARC insertion

Authors response: Dear reviewer, thank you for your comments, this has been addressed, kindly see line 92.

Reviewer comments: Line 93- 95: Few studies have been conducted on LARC methods [4, 15, 16], however, majority of these focused on adolescent and reproductive-age women.

Authors response: Thank you for your observations, this has been corrected accordingly, kindly see line 94-95.

Reviewer comments: Line 135: Of the community level factors, residence (rural, urban) were directly accessed from the EDHS data set. Full term required here for EDHS

Authors response: Thank you for your comments, this has been addressed, kindly see line 131-132.

Reviewer comments: Results are interpreted adequately.

Authors response: Thank you

Reviewer comments: Line 183: postpartum women also used LARC if they had institutional delivery.

Authors response: Thank you for your suggestions, this has been addressed, kindly see line 185-186.

Reviewer comments: First paragraph should be reduced with minimum repetitions.

Authors response: thank you for your comments and suggestions, this has been corrected accordingly, kindly see line 215-217.

Reviewer 2

Reviewer comments: This is a very well written article on the important topic of postpartum LARC use and factors that contribute to the use of LARC in Sub Saharan African countries. The topic chosen is very important and addresses one of the essential strategies to meet the 2030 SGD of reducing MMR to below 70/100000. The data has been taken from the DHS data base, thereby including a large population form different Sub Sahran countries with high MMR. Important factors like socioeconomic status, educational status, ANC visits PNC visits etc that can affect use of LARC have been touched upon. The discussion is appropriately argued with respect to other studies.

Conclusion is aligned with the results.

Authors response: Thank you
